# Targeted mechanical forces enhance the effects of tumor immunotherapy by regulating immune cells in the tumor microenvironment

**DOI:** 10.20892/j.issn.2095-3941.2022.0491

**Published:** 2023-01-12

**Authors:** Pengfei Zhu, Hongrui Lu, Mingxing Wang, Ke Chen, Zheling Chen, Liu Yang

**Affiliations:** 1 Cancer Center, Department of Medical Oncology, Zhejiang Provincial People’s Hospital (Affiliated People’s Hospital, Hangzhou Medical College), Hangzhou 310014, China; 2 Graduate School of Clinical Medicine, Bengbu Medical College, Bengbu 233000, China; 3 Department of Gastroenterology & Pancreatic Surgery, Zhejiang Provincial People’s Hospital, Hangzhou 310014, China

**Keywords:** Mechanical force, microenvironment, immunotherapy, extracellular matrix, cancer

## Abstract

Mechanical forces in the tumor microenvironment (TME) are associated with tumor growth, proliferation, and drug resistance. Strong mechanical forces in tumors alter the metabolism and behavior of cancer cells, thus promoting tumor progression and metastasis. Mechanical signals are transformed into biochemical signals, which activate tumorigenic signaling pathways through mechanical transduction. Cancer immunotherapy has recently made exciting progress, ushering in a new era of “chemo-free” treatments. However, immunotherapy has not achieved satisfactory results in a variety of tumors, because of the complex tumor microenvironment. Herein, we discuss the effects of mechanical forces on the tumor immune microenvironment and highlight emerging therapeutic strategies for targeting mechanical forces in immunotherapy.

## Introduction

Tumors are characterized by uncontrolled growth, avoidance of apoptosis, and resistance to antitumor drugs^1^. To date, the treatment options for tumors include surgery, chemotherapy, radiotherapy, targeted therapy, and immunotherapy^2^. Tumor cells and the tumor microenvironment (TME) play key roles in tumor progression and drug efficacy^3,4^. Tumor cells and their surrounding immune cells, stroma, tumor angiogenesis, and biomolecules constitute a complex TME^4^. Although immunotherapy has achieved therapeutic effects in a variety of solid tumors, such as lung cancer, lymphoma, melanoma, and kidney cancer^5–8^, many solid tumors have low immunogenicity, and consequently immunotherapy has poor efficacy. Therefore, a need exists to analyze various aspects of the bottleneck in tumor immunotherapy. Many tumor immunotherapy studies have focused on genetic and biochemical processes, whereas physical factors have often been ignored. Tumor cells are usually confined to a specific microenvironment, such as the extracellular matrix (ECM), whose changes affect the behavior of tumor cells^9^. Consequently, the roles of the mechanical properties of the microenvironment in immunotherapy are worthy of attention. In this review, we discuss how the TME and its characteristics affect tumor progression, invasion, and metastasis. The effects of mechanical forces on various immune cells in the TME are analyzed with the aim of improving the effects of tumor immunotherapy on the basis of targeted mechanical force.

## Mechanical properties of the TME: the tumor mechanical microenvironment

**Figure 1:**
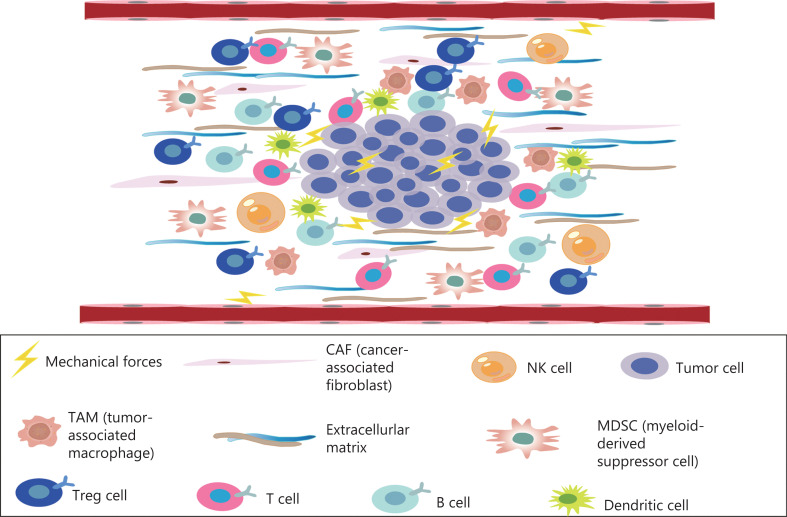
The TME, a complex mixture of tumor cells, stromal cells, tumor microvessels, a variety of immune cells, cancer related fibroblasts, and non-cellular components in the ECM—is closely associated with the occurrence, growth, and metastasis of malignant tumors.

The TME comprises a complex mixture of tumor cells, stromal cells, tumor microvessels, multiple immune cells, carcinoma-associated fibroblasts, and non-cellular components within the ECM (**Figure 1**)^9^. The TME plays important roles in the occurrence, progression, metastasis, and therapeutic responses of tumors^10^. The TME is characterized by hypoxia, chronic inflammation, and immunosuppression^11^, thus creating an environment that is highly conducive to tumor proliferation, invasion, adhesion, and angiogenesis, and induces resistance to radiation and chemotherapy, thereby promoting the occurrence of malignant tumors^12^. The microenvironment of tumor tissue differs from that of normal tissue, mainly in its abnormal vascular and lymphatic structure and function, high interstitial pressure, and dense interstitial matrix. Recent studies have highlighted that, in addition to biochemical signals from the microenvironment, physical signals significantly alter cell behavior, such as proliferation, metastatic potential, and the characteristics of cancer stem cells. Physical signals in tumors comprise primarily 3 aspects: increasing matrix hardness, solid stress, and interstitial hydraulic pressure^13,14^. The mechanical properties of the TME are also involved in the regulation of tumor growth, invasion, and metastasis. Changes in tissue mechanics often promote disease progression by altering cell behavior^15,16^. Mechanical forces do not act independently in tumors, but instead act in conjunction with the development and progression of tumors. Tumor growth requires the generation of mechanical forces both within the tumor and between the tumor and the host tissue, thus resulting in abnormal solid and liquid stresses^14^. The occurrence of solid stress plays important roles in tumor cell genetic changes, tumor cell proliferation, tumor cell invasiveness, and anti-tumor drug resistance^17–21^. In solid tumors, mechanical force is caused by an increase in structural composition, particularly the number of cancer cells, stromal cells, and ECM components^14^. ECM remodeling and sclerosis are important features of solid tumors^22^. The overproduction of ECM is associated with the occurrence of liver cancer caused by chronic liver disease. The measured hardness of liver cancer tissue is approximately 10 times that of normal liver tissue^22,23^. In addition, pancreatic ductal adenocarcinoma (PDAC) has a highly fibrotic matrix composed primarily of ECM and cancer-associated fibroblasts^24,25^. In the pancreatic cancer TME, continuous ECM remodeling is characterized by the continuous degradation and deposition of ECM molecules, such as collagen^26^. This extensive collagen deposition in the TME increases tumor density and thus alters its mechanical properties with respect to those of normal pancreatic tissue^27^. These changes in turn change the mechanical properties of the TME.

## Mechanical properties of the ECM and tumor fate

**Figure 2:**
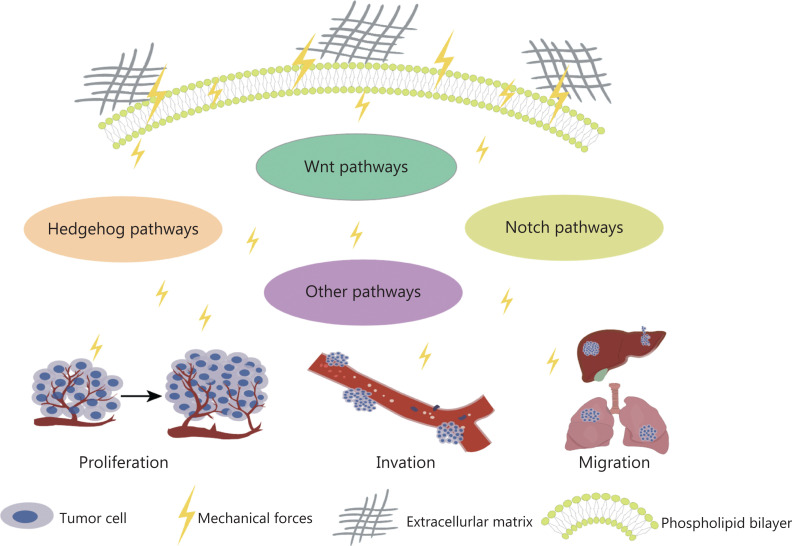
Mechanical force activates signaling pathways, thus promoting tumor cell proliferation, driving invasion, and enhancing cell migration.

Various tissues and organs have different biomechanical properties, as do tumor tissues^28^. The mechanical properties of the ECM play key roles in determining cell fate and are driven largely by internal cytoskeletal changes that alter cell tension. Cells can sense their physical environment or ECM in a process known as “mechanosensing”^29^. Overall, the ECM is composed of fibrous proteins, such as collagen, proteoglycans, glycosaminoglycans, elastin, fibronectin, and laminin, which are controlled by the ECM and provide mechanical support to cancer cells^30^. Their proportions, post-translational modifications, degree of cross-linking, and arrangement determine their organizational nature^31^. Physical interactions between cells and the ECM influence many cellular behaviors associated with cancer progression through the Notch, Wnt, and Hedgehog pathways (**Figure 2**)^32^. The mechanical force increases with tumor growth, thus making cancer cells invasive^19^. The interaction between cells and the ECM depends on focal adhesion and cytoskeletal proteins, which regulate cell shape and motion^33^. In addition, mechanical forces are another major factor regulating cytoskeletal dynamics and cell survival^34^. In tumors, the mechanical forces of attached cells are derived primarily from the biophysical properties of the ECM and intertissue fluid pressure, whereas the main mechanical forces in detached cells arise from hydrodynamics, such as fluid shear flow^35^. Both ECM contraction and increased mechanical force caused by fluid shear flow can induce apoptosis^36^.

## Mechanical properties of the ECM and the tumor immune microenvironment

**Figure 3:**
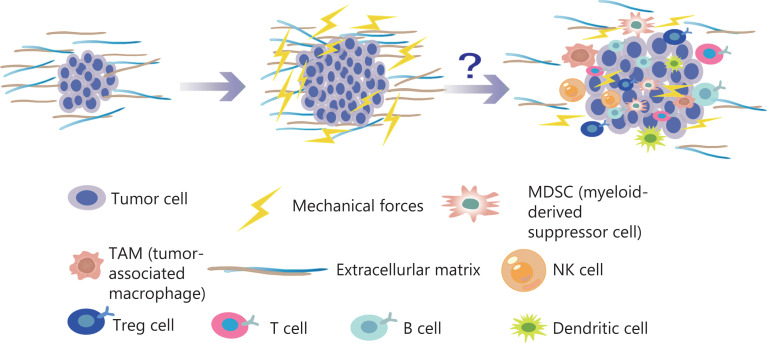
With an increase in the number of tumor cells and the reconstruction of ECM, the pressure inside the tumor gradually increases. The mechanical force continues to increase, thus generating a pressure load on each cell in the TME. Many studies have found that the generation of mechanical force has substantial effects on tumor proliferation, invasion, and metastasis. Similarly, mechanical force influences a variety of immune cells in the TME.

Among the novel immune therapies for tumors, immune checkpoint inhibitors are an effective treatment^37^. Remodeling of the ECM modulates the immune system, thus leading to disorders in the immune response. Specifically, immune cells actively participate in the regeneration of injured tissue and promote the deposition and formation of ECM. In turn, the ECM contributes to the development of tumor immunosuppression. Cytokines and chemokines secreted by immunosuppressive networks can lead to tumor immune escape^38–43^. In addition, in the pancreatic cancer microenvironment, ECM protein deposition increases tissue tension and intratumor pressure, thus leading to increased hypoxia and resulting in immunosuppression^44^. In the tumor immune microenvironment, immune cells are the main cellular components, primarily T cells, B cells, mononuclear macrophages, natural killer cells (NK), and their subgroups^45^. In a preclinical study, lysyl oxidase inhibition has been found to have different mechanical regulatory effects depending on the structure of the ECM, and to significantly increases T cell mobility^46^. Previous studies have shown that malignant transformation of tumor cells and tumor progression are associated with cancer cell softening^47^. Studies have shown that epithelial mesenchymal transition sclerosis is associated with the invasion and metastasis of breast cancer^48^. High hardness of tumor tissue enhances epithelial mesenchymal transition, over-activates signaling pathways, promotes cancer-endothelial interactions, drives invasion, enhances cell migration, and prevents immune cells from infiltrating into the TME^49–56^. With increases in the numbers of tumor cells and non-cancer cells, and ECM remodeling, the pressure inside tumors gradually increases, and the resulting strong mechanical forces continually produce a pressure load on each cell in the TME. As described above, many studies have found that the generation of mechanical force greatly affects tumor proliferation, invasion, and metastasis; however, how the mechanical forces affect the efficacy of tumor cell immunotherapy is unclear (**Figure 3**).

### Mechanical forces and T cells

**Figure 4:**
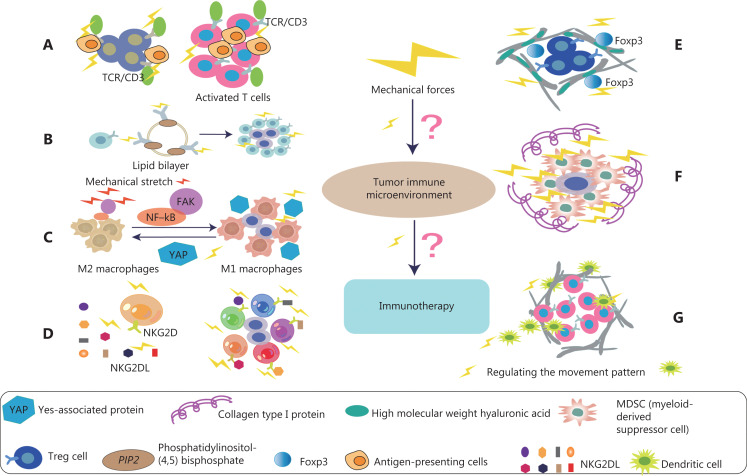
Effects of mechanical forces on various immune cells in TME. (A) The activation of T cells depends on the adhesion of T cells to antigen presenting cells (APC). In addition, the mechanical force exerted on the T cell receptor (TCR) may promote a conformational change in TCR/CD3 complex, thus activating T cells and signaling pathways. (B) B cell receptors (BCRs) sense the chemical and physical characteristics of antigens. Mechanical force induces more sensitive to the activation of IgG-BCR–expressing memory B cells. This mechanism is achieved by enriching the important signal transduction phospholipid PI(4,5)P_2_ through the positively charged IgG tail of the plasma membrane tethered in quiescent B cells, thus promoting the activation of IgG BCR. (C) Mechanical stretching (MS) enhances M1 polarization and anticancer effects. Specifically, MS promotes tumoricidal polarization of M1 macrophages through the FAK/NF-κB signaling pathway. In addition, YAP senses the hardness of the surrounding matrix and stimulates macrophages to transform into the immunosuppressive M2 type, which secretes many immunosuppressive factors. (D) In NK cell antitumor immunotherapy, one of the most relevant NK cell activation receptors is NKG2D, which recognizes 8 different NKG2D ligands (NKG2DL). NKG2D, under the influence of mechanical forces, can distinguish among ligands and thus induce different NK activities. (E) The complete ECM component high molecular weight hyaluronic acid (HMW-HA) has a role in actively maintaining immune tolerance. HMW-HA promotes *in vitro* persistence of Foxp3, a key transcription factor for Tregs. HMW-HA also actively promotes the function of CD4+CD25+ Tregs. (F) Col1 associates with tumor tissue, thus generating a biophysically “rigid” microenvironment around cancer cells that promotes cell migration, and induces cancer cell proliferation and survival. High collagen deposition in the TME increases tumor density and thus alters mechanical properties. These alterations increase solid stress, interstitial fluid pressure, and hardness in tumors, and together lead to changes in tissue microstructure. (G) The fibroblast matrix and the associated ECM around the tumor provide physical constraints on infiltrating DCs. Mechanical forces are ubiquitous in organisms, and fluid shear stress regulates the movement patterns of DCs.

The ability of T cells to recognize and kill cancer cells has become a focus of research on immune-based cancer therapies. In most tumors, however, T cells are often dysfunctional. The TME is the main factor affecting the normal function of T cells^57^. The immunological synapse formed between a cytotoxic T lymphocyte and an infected or transformed target cell is a physically active structure capable of exerting mechanical force^58^. Studies have shown that the PI3K-DOCK2 signal acts on cytotoxic T lymphocytes and drives cellular mechanical forces at the interface perpendicular to the cell surface^58^. In addition, enhanced cytotoxicity to sclerotic cancer cells is mediated by enhanced T cell force, owing to increased accumulation of filamentous actin at immune synapses. Activation of T cell signals is largely dependent on T cell adhesion to antigen-presenting cells as well as extensive rearrangement of the actin cytoskeleton and cell deformation. In addition, physical forces applied to the T cell receptor (TCR) may promote activation by promoting conformational changes in the TCR/CD3 complex (**Figure 4A**), thus leading to signal activation, TCR clustering, and signal micro-cluster assembly^59–61^. The combination of lysyl oxidase inhibition and PD-1 blocking therapy increases the accumulation of effector CD8 T cells in tumors and significantly delays tumor progression^46^. YES-associated protein (YAP) is a mature nuclear transcription coactivator that responds to a variety of mechanical changes, including ECM hardness, cellular structure, and cytoskeletal alterations. YAP is the best-known microenvironmental mechanical sensor. In the TME, YAP enhances sensitivity to external mechanical forces, affects and destroys adjacent ECM, and inhibits the metabolic reprogramming of effector T cells^62,63^. Recent studies have shown that tumor cells have a cholesterol-rich plasma membrane, and that hardening of cancer cells by cholesterol depletion enhances the cytotoxicity of T cells and improves the efficacy of adoptive T cell therapy against solid tumors in mice^64^.

### Mechanical forces and B cells

Current immunotherapies are designed to activate killer T-cell immune cells to combat cancer, but only 20 percent of patients experience lasting clinical benefits from this treatment^65^. Increasing studies indicate that T cells are not the only cells involved in immunotherapy. Many other types of immune cells are involved in this process, including B cells^66^. B cells and tertiary lymphoid structures play important roles in immune checkpoint blockade therapy^66^. B cell-associated genes, such as *MZB1*, *JCHAIN*, and *IGLL5*, are significantly more highly expressed in patients who have responded to immunoadjuvant therapy for melanoma than in patients who have not responded to immunotherapy before treatment^67,68^. Thus, in early stages of immunotherapy, the density of CD20-positive B cells, tertiary lymphatic structure, and the ratio of tertiary lymphatic structure to tumor area are higher in patients who respond to immune checkpoint inhibitors than in those who do not respond to treatment. Multiple immunofluorescence analysis has indicated that CD20-positive B cells are located in the tertiary lymphoid structure of tumors and co-localize with CD4-positive T cells, CD8-positive T cells, FOXP3-positive T cells, and CD21-positive follicular dendritic cells (DCs). B lymphocytes use B cell receptors (BCRs) to sense the chemical and physical characteristics of antigens^69^. Research has shown that mechanical force induces more sensitive to the activation of IgG-BCR–expressing memory B cells. This mechanism is achieved by enriching the important signal transduction phospholipid PI(4,5)P_2_ through the positively charged IgG tail of the plasma membrane tethered in quiescent B cells, and promoting the activation of IgG BCR with a low mechanical force threshold^69^ (**Figure 4B**). Diacylglycerol kinases terminate diacylglycerol signaling and promote phosphatidic acid production^70^. Recent results have suggested that diacylglycerol kinase ζ shapes B cell responses by modulating actin remodeling, force production, and antigen uptake related events in immune synapses^71^. Therefore, exploring the response of mechanical forces to B cells is likely to be highly valuable in regulating tumor immunotherapy in the future.

### Mechanical forces and macrophages

Macrophages are immune cells that reside in tissues and play key roles in maintaining homeostasis and fighting against infection^72^. Macrophages are crucial in the progression of pathophysiological conditions such as cancer, cardiovascular disease, obesity, wound healing, and foreign body reaction. In cancer, for example, M1 macrophages have tumoricidal functions, whereas M2-like macrophages help tumor cells evade host immune cell destruction and promote angiogenesis, invasion, and metastasis. Most tumor-associated macrophages have an M2-like phenotype, and the presence of macrophages in tumors is directly associated with poor prognosis^73,74^. Macrophages may be a part of tumor-cell-based cancer immunotherapy. The polarization of macrophages is crucial in the antitumor process. Previous studies have shown that mechanical stretching (MS), an abiotic modulated method, enhances M1 polarization and anticancer effects^75^. Specifically, MS promotes tumoricidal polarization of M1 macrophages through the FAK/NF-κB signaling pathway^75^ (**Figure 4C**). In addition, YAP senses the hardness of the surrounding matrix and stimulates macrophages to transform into the immunosuppressive M2 type, which secretes many immunosuppressive factors^76^. *In vivo* and *in vitro* studies have shown that macrophages are mechanically reactive, and importantly alter their polarization state in response to surrounding mechanical stimuli^77^. However, in tumor immunotherapy, the specific mechanism through which mechanical force acts on their polarization state remains unclear. In the TME, enhancing immunotherapy by targeting mechanical force and promoting the M1 transformation of macrophages is important.

### Mechanical forces and NK cells

NK cells are cytotoxic lymphocytes in the innate immune system, which have been shown to kill cancer cells and play an important role in tumor immunotherapy^78^. NK cells are susceptible to a variety of immunosuppressive mechanisms that are active in the TME. The actin cytoskeleton plays crucial roles in a variety of cellular processes. The diversity and flexibility of lymphocyte function are facilitated by the formation of various actin structures that provide the mechanical forces required for the secretion, movement, adhesion, and tissue invasion of lysed particles^79,80^. A growing body of evidence strongly suggests that dynamic actin networks, rather than static networks, are critical for regulating cellular responses^81,82^. Recent studies have demonstrated that actomyosin retrograde flow (ARF), a major regulator of the NK cell immune response, alters the NK cell configuration state through an interaction between β-actin and SH2-domain protein tyrosine phosphatase-1 (SHP-1)^83^. In NK cell antitumor immunotherapy, one of the most relevant NK cell activation receptors is NKG2D, which recognizes 8 different NKG2D ligands (**Figure 4D**). NKG2D binds its ligands and subsequently triggers the function of NK cell effectors^84^. Recent results have indicated that NKG2D, under the influence of mechanical forces, can distinguish among ligands and thus induce different NK activities^85^. Overcoming and regulating the adverse tumor mechanics and regulating the ability of immune receptor recognition may further improve the effects of immunotherapy and provide new ideas for future therapeutics.

### Mechanical forces and regulatory T cells

Regulatory T cells (Tregs) are a class of immune regulatory cells that induce immune tolerance. They maintain the balance between immune cells by controlling and coordinating the immune responses of effector T cells, mast cells, DCs, and B cells *in vivo*. In the TME, Tregs exert immunosuppressive effects mainly by inhibiting cell–cell contact, expression of surface molecules, and secretion of cytokines. The high molecular weight hyaluronic acid (HMW-HA), which is the main component of ECM, plays an active role in maintaining immune tolerance. HMW-HA promotes *in vitro* persistence of Foxp3, a key transcription factor for Tregs. HMW-HA also actively promotes the function of CD4+CD25+ Tregs^86^ (**Figure 4E**). Liver fibrosis is the cause of abnormal accumulation of ECM and ineffective removal of fibrosis^87^. Recent studies have shown that many Tregs are dispersed around sites of fibrous hyperplasia, and the expansion of Tregs promotes the regression of liver fibrosis. In addition, the expression of matrix metalloproteinases and tissue inhibitors of metalloproteinases is altered by Treg depletion, and the persistence of cirrhosis is maintained by increased numbers of Tregs at fibroproliferative sites and subsequent regulation of the balance between matrix metalloproteinases and tissue inhibitors of metalloproteinases^87^.

### Mechanical forces and myeloid-derived suppressor cells

Myeloid-derived suppressor cells (MDSCs) are pathologically activated neutrophils and monocytes with strong immunosuppressive activity. MDSCs consist of 2 large groups of cells: granulocyte or polymorphonuclear MDSCs and monocyte MDSCs^88^. Type I collagen (Col1) has been reported by many investigators to be an important component of the ECM deposited in the PDAC matrix^89,90^. Col1 associates with tumor tissue, thus generating a biophysically “rigid” microenvironment around cancer cells that promotes cell migration by favoring Col1 fiber “tracks,” and promoting aberrant cellular interactions that induce cancer cell proliferation and survival^51,91,92^ (**Figure 4F**). In pancreatic malignancies, substantial collagen deposition in the TME increases tumor density and thus alters the mechanical properties of tumor tissue with respect to those in normal pancreatic tissue. These alterations increase solid stress, interstitial fluid pressure, and hardness in tumors, and together lead to changes in tissue microstructure^27^. Recent studies have shown that activated pancreatic stellate cells/αSMA and myofibroblasts are the major contributors of Col1 in the PDAC matrix. Deletion of Col1 in myofibroblasts leads to upregulation of CXCL5 *via* SOX9 in cancer cells. Increased CXCL5 is associated with recruitment of bone MDSCs and inhibition of CD8+ T cells^93^. Therefore, the increase in Col1 deposition might possibly lead to changes in mechanical force in the TME, which have important effects on the recruitment of MDSC. How the above cellular components are regulated by mechanical force to affect the mechanism of immunotherapy is still unclear.

### Mechanical forces and DCs

DCs, the most powerful professional antigen-presenting cells in the immune system, play key roles in the initiation and regulation of the immune response. According to their origin, 2 main groups can be distinguished: plasmacytoid DCs and myeloid DCs (conventional DCs, mDCs, or cDCs)^94^. DCs are the initiator of the body’s immune response, and their primary characteristic is stimulation of primary T cell proliferation. The fibroblast matrix and the associated ECM around the tumor provide physical constraints on infiltrating DCs, and regulate DC maturation and trafficking, thereby influencing T-cell function^95^. Myelofibrosis is believed to promote the pathological remodeling of the ECM, and studies have confirmed that the physical and mechanical properties of myelofibrosis contribute to the abnormal differentiation of monocytes, thus causing proinflammatory polarization of monocytes and differentiation into DCs^96^. Mechanical forces are ubiquitous in organisms that activate DCs, which subsequently migrate to inflammatory sites in draining lymph nodes. Fluid shear stress regulates the movement patterns of DCs (**Figure 4G**). DCs with shear stress show elevated expression of the DC activation markers MHC class I and CD86^97^. Biomechanical forces thus play important roles in regulating DC migration and activity.

In summary, we described the characteristics of the tumor mechanical microenvironment arising from the mechanical properties generated through the interaction of tumor cells and ECM. The tumor mechanical microenvironment has different degrees of influence on various immune cells in the tumor immune microenvironment (**Figure 4**). This relationship may guide immunotherapy in clinical practice in the future.

## Prospects for the clinical application of mechanical force in tumor immunotherapy

Immunotherapy has been a mainstay of treatment for advanced solid tumors, but for some “cold” tumors, immunotherapy does not appear to achieve satisfactory therapeutic effects, mainly because of the lack of general tumor-specific antigens, and the immunosuppressive TME’s inhibition of lymphocyte infiltration and activation. Abnormal vascular distribution, a characteristic of malignant solid tumors, promotes the formation of a immunosuppressive microenvironment and induces tumor resistance to immunotherapy. Recent clinical studies have found that the hardness of liver tumor tissue in patients with colorectal cancer with liver metastasis is higher than that of liver tissue without liver metastasis^98^. Shen et al.^98^ have inhibited fibroblast contraction and ECM deposition by targeting the renin angiotensin system, thereby decreasing liver hardness and increasing the anti-angiogenic effect of bevacizumab. The study has reported that patients who received bevacizumab together with renin angiotensin inhibitor treatment achieved longer survival^98^. The combination of anti-angiogenic therapies with immunotherapy may offer exciting opportunities for the treatment of solid tumors^99–101^. The influence of tumor matrix stiffness on tumor immunotherapy remains to be studied from a macroscopic perspective. Moreover, from a microscopic perspective, the generation or elimination of physical and mechanical forces in the TME and the specific mechanism of immunotherapy must be further explored. Whether mechanical therapy can further improve therapeutic effects through a combination of advanced tumor immunotherapy and targeted vascular therapy must be better understood.

In the immunotherapy of malignant tumors, T lymphocytes are the main actors in the response to immunotherapy^102^. Strikingly, cancer immunotherapy produces a curative response in a small percentage of patients with relapsed or refractory cancer; however extending these clinical benefits to most cancer patients remains challenging^103^. In tumor immunotherapy, T cells are gradually depleted, and how to replenish T cells remains an open question^104^. At present, combined immunotherapy has a positive effect on the activation of dysfunctional T cells. For example, when PD-1 and TIGIT are blocked in combination, CD8+ TILs cultured in autologous tumors show an additive effect in restoring effector function^105^. In terms of biomechanics, T cells continually experience different biomechanical environments throughout their life cycle, such as shear forces in the bloodstream and extensive tissue stiffness. Biomechanics, including passive and active mechanical forces, has been shown to control T cell development, activation, migration, differentiation, and effector function. From a biomechanical viewpoint, recent studies by Tang et al.^106^ have attempted to optimize T cell activation and expansion by applying force to T cell populations. Tang et al.^64^ have found that cancer cells are stiffened by depletion of their membrane cholesterol, thus enhancing T-cell vitality and cytotoxicity by overcoming mechanical immune checkpoints. Restoring the activity of tumor-specific T cells greatly improves their clinical effects against cancer. In this review, we discussed current knowledge regarding the effects of biological mechanical forces and the understanding of the dynamic recovery of T cell dysfunction in cancer. With this new understanding, we believe that research on mechanical force and the energy recovery mechanisms of specific immune cells would be valuable and should not be ignored.

## Conclusion

Tumor immunotherapy is currently a clinical research hotspot. However, many difficulties remain to be overcome in its application to solid tumors. Therefore, this review considered a variety of factors for increasing the efficacy of tumor immunotherapy. Mechanical force is an easily overlooked factor in tumor treatment. In this review, we described the mechanical characteristics of the tumor immune microenvironment, and the mechanical influence of a variety of immune cells in the TME, and discussed interactions between the mechanical environment and the immune microenvironment. These findings may open new doors to future clinical applications.

In addition, we discussed various clinical applications of mechanical force in tumor immunotherapy. However, many studies on the effects of mechanical forces on tumor therapy have remained in preliminary stages, and the specific interaction mechanisms of force generation between ECM and tumors, and the links between mechanical forces and immune cells, remain to be clarified. Exploring how mechanical force can be used to improve the outcome of tumor immunotherapy is crucial.

In the future of cancer treatment, several problems remain to be solved. We believe that the magnitude of the mechanical forces produced varies across tumors, and for the same type of tumor, differences will exist among individuals and in different populations. Currently, a precise method to measure the mechanical forces inside tumors is lacking. The influence of the distribution structure and proportion of mechanical forces in the TME on tumor proliferation, metastasis, and drug resistance remains to be explored.
